# Robust expression of EZH2 in endocervical neoplastic lesions

**DOI:** 10.1007/s00428-019-02550-8

**Published:** 2019-03-22

**Authors:** Evelin Makk, Levente Bálint, János Cifra, Tamás Tornóczky, Angéla Oszter, Arnold Tóth, Endre Kálmán, Krisztina Kovács

**Affiliations:** 1 Department of Pathology, University of Pécs Medical School, Szigeti út 12, Pécs, 7624 Hungary; 2 Department of Pathology, County Hospital Tolna, János Balassa Hospital, Béri Balogh Ádám u. 5-7, Szekszárd, 7100 Hungary; 3 Department of Radiology, University of Pécs Medical School, Ifjúság út 13, Pécs, 7624 Hungary

**Keywords:** Cervical cancer, EZH2, Endocervical adenocarcinoma, AIS

## Abstract

The aim of this study was to evaluate the nuclear expression of histone methyltransferase enhancer of zeste homolog 2 (EZH2) in endocervical neoplastic lesions such as invasive endocervical adenocarcinoma (ECA) and cervical in situ adenocarcinoma (AIS) in comparison with normal endocervix and non-neoplastic counterparts. A total of 54 consecutive neoplastic cases (37 ECA, 17 AIS) and 32 non-neoplastic endocervical lesions (15 reactive atypia, 9 microglandular hyperplasia, 3 tuboendometrioid metaplasia, 3 tunnel cluster, 2 endometriosis) were included in the study with adjacent normal endocervix if present. EZH2 immunoreactivity was evaluated semiquantitatively by three independent experts in lesions and adjacent normal glandular epithelium as well. EZH2 expression was defined robust if at least two of the three experts rated partial or diffuse positivity. Robust EZH2 expression was statistically compared among the neoplastic, non-neoplastic, and normal glandular epithelium samples. Diagnostic test capability of robust EZH2 expression was calculated. Fifty-three out of the 54 neoplastic cases (98%) showed robust EZH2 expression. Robust EZH2 expression was significantly less often (4 out of 32 cases, 12.5%) found in the non-neoplastic endocervical lesions (*p* < 0.0001) and never (0 out of 66 samples) in the adjacent normal glandular epithelium. Robust EZH2 overexpression had a sensitivity and specificity of over 95% in detecting neoplastic lesions versus non-neoplastic lesions or normal glandular epithelium. EZH2 may play a role in the pathogenesis of endocervical neoplasia, and the detection of robust expression of EZH2 might be a useful differential diagnostic tool in problematic endocervical lesions in histology and cytology as well.

## Introduction

Endocervical adenocarcinoma (ECA) is the second most common histological type of cervical cancer; it comprises approximately 20 to 25% of cervical malignancies [1] and has a poorer prognosis than squamous cell carcinoma [2]. Cervical adenocarcinomas and their precursor lesions are heterogeneous and have several different subtypes, most of them closely related to HR-HPVs [3].

It is well established that the pRB pathway is involved in the pathogenesis of cervical cancer due to the interaction with HR-HPV E7 oncoproteins leading to genomic instability [4]. It is also known that viral E6/E7 oncoproteins may interact with different types of epigenetic enzymes, such as p300, CBP, and pCAF, which can be involved in the oncogenesis [5].

Enhancer of zeste homolog 2 (EZH2), a member of the polycomb group of genes, is a methyltransferase that methylates histone H3 on gene promoters and plays a critical role in epigenetic gene silencing and chromatin remodeling. EZH2 inhibits cell differentiation and targets gene expression. In conjunction with the p53 protein, it induces tumor cell proliferation, metastasis, and immortalization [6].

Recent studies focused on the role of EZH2 in the pathogenesis of various adenocarcinomas as well as malignant tumors of the breast [7], lung [8], stomach [9], colon [10], pancreatobiliary tract [11], liver [12], thyroid gland [13], prostate [14], bladder [15], uterus [16], and ovary [17]. High expression of EZH2 was shown to be associated with tumor aggressiveness and was suggested as a potential differential diagnostic marker [8, 10–17]. In the cervix, one study reported overexpression and the possible prognostic significance of EZH2 in squamous cell carcinoma [18].

Expression of EZH2 in endocervical neoplastic lesions is yet unknown. In this study, we examined EZH2 expression in ECA and AIS, compared with non-neoplastic cervical lesions and normal glandular epithelium.

## Materials and methods

### Patients and specimen collection

Consecutive patients from 2007 to 2017 with a diagnosis of endocervical neoplastic lesions (ECA and/or AIS with or without cervical intraepithelial neoplasia) and patients with benign findings as a control group were collected from the archives of the Department of Pathology, University of Pécs, Hungary, and Department of Pathology, County Hospital Tolna, János Balassa Hospital, Szekszárd, Hungary.

Formalin-fixed and paraffin-embedded tissue samples from biopsy, cone, or hysterectomy specimens were available along with the HE slides in each case. Slides were re-evaluated to select the most feasible specimens for immunohistochemistry for each patient. We classified endocervical adenocarcinomas based on the International Endocervical Adenocarcinoma Criteria and Classification (IECC) [19].

This work has been approved by the local ethical committee (number of permission: PTE/57682/2017).

### Immunohistochemistry

Prior to immunohistochemistry, formalin-fixed paraffin-embedded tissue specimens were cut into 4-μm-thick sections and dried for 20 min at 60 °C.

Immunostaining was performed using Leica Bond Max autostainer (Leica Biosystems, Bannockburn, IL) and Leica Bond Polymer Refine Detection Kit (Leica Biosystems, Newcastle Upon Tyne, UK). The mouse monoclonal EZH2 antibody (clone 6A10) was obtained from Leica Biosystems (Newcastle Upon Tyne, UK) and used at a dilution of 1:200. The immunostaining protocol included deparaffinization and pH 9 epitope retrieval for 20 min, peroxidase blocking for 5 min, primary antibody incubation for 15 min, post-primary rabbit anti-mouse IgG for 8 min, polymer anti-rabbit Poly-HRP-IgG for 8 min, diaminobenzidine chromogen for 10 min, and hematoxylin counterstain for 5 min. Positive and negative controls were included in all reactions.

### Evaluation of immunoreactivity

Immunoreactivity evaluation included not only the neoplastic lesions (ECA, AIS) or non-neoplastic lesions in control patients but the adjacent normal glandular epithelium as well, if present. The presence of concurrent cervical intraepithelial neoplasia was noted; however, these lesions were not included in the immunoreactivity analysis. The histological patterns were detected in the original, routine HE stained slides.

Immunoreactivity was evaluated semiquantitatively by three independent board-certified pathologists with over 15 years of professional experience (expert 1: E.K., expert 2: K.K., expert 3: A.O.). Cases were regarded as positive if they were obviously positive by × 40 magnification and further classified according to the percentage of cells with nuclear staining: < 10% as focally positive “+”, 10–50% as partly positive “++”, and > 50% as diffusely positive “+++” [20].

The inter-expert agreement was determined using Intraclass Correlation Coefficient (ICC) [21] for both the neoplastic and the non-neoplastic lesions ratings. Two-way model, absolute agreement type was applied; both single and average measurement reliability was calculated. The analysis was run in MedCalc statistical software (version 13.0.0.0, MedCalc Software bvba, Ostend, Belgium) [22].

For statistical analyses, the individual ratings per lesions and normal glandular epithelium if present were transformed into a binary overall score. Immunoreactivity of a lesion or normal glandular epithelium was regarded “robust” if at least two of the three experts rated either “++” or “+++”. Immunoreactivity was regarded as “negative/focally positive” if at least two of the three experts rated either “−” or “+”. Adjacent normal glandular epithelium was included in analyses if it was detected by at least two experts.

Neoplastic (ECA and AIS) and non-neoplastic lesion immunoreactivity overall scores were statistically compared using Fisher’s exact test (MedCalc). *P* value was considered statistically significant if under 0.05.

Diagnostic test capability (sensitivity, specificity, and positive and negative predictive value) of EZH2 overexpression in distinguishing (a) neoplastic lesions from non-neoplastic, (b) neoplastic lesions from normal endocervical epithelium, and (c) neoplastic lesions from non-neoplastic lesions and normal endocervical epithelium combined was evaluated also using MedCalc.

## Results

A total of 54 cases of endocervical neoplastic lesions (37 ECA, 17 AIS) were retrieved from the archive. In 12 out of these cases, concurrent HSILs were present. Concurrent LSIL was present in one case. The median patient age was 44.5 (range 29–84). The most common IECC diagnoses were human papillomavirus–associated adenocarcinoma (HPVA) type (92% of the cohort). Between subcategories, usual type adenocarcinoma was the most common HPVA type (88% of the cohort), followed by villoglandular, mucinous not otherwise specified (NOS), and mucinous including intestinal and invasive stratified mucin-producing carcinoma (iSMILE) categories (3%) (Table 1). There were only three patients with nonhuman papillomavirus–associated adenocarcinoma (NHPVA) (8%). Between subcategories, there were two cases with serous type and one with the endometrioid type of NHPVA.

**Table 1:** Clinical and immunostaining data of ECA cases

Case No.	Age	Diagnosis	Type (IECC^4^)	EZH2 immunoreactivity^3^							
				Normal glandular epithelium				Neoplastic endocervical lesions			
				Expert			Overall score^9^	Expert			Overall score^9^
				1	2	3		1	2	3	
1.	83	ECA	HPVA^5^/mucinous, intestinal	ø^2^	ø	ø	ø	+++	+++	+++	+
2.	84	ECA	HPVA/usual	–	ø	–	–	+++	+++	+++	+
3.	74	ECA	HPVA/usual	ø	ø	ø	ø	+++	+++	++	+
4.	53	ECA + CIN3^1^	HPVA/usual	–	+	–	–	+++	+++	+++	+
5.	50	ECA	NHPVA^6^/endometrioid	–	–	–	–	+	+	++	**−**
6.	48	ECA	HPVA/usual	ø	ø	–	ø	+++	++	+++	+
7.	49	ECA + CIN3	HPVA/usual	ø	ø	ø	ø	+++	+++	+++	+
8.	48	ECA	HPVA/usual	ø	ø	–	ø	+++	++	++	+
9.	42	ECA + AIS	HPVA/usual	+	–	–	–	+++	+++	+++	+
10.	45	ECA	HPVA/usual	–	–	–	–	+++	+++	+++	+
11.	37	ECA	HPVA/usual	–	–	–	–	+++	+	+++	+
12.	41	ECA	HPVA/villoglandular	ø	ø	ø	ø	+++	+++	+++	+
13.	37	ECA	HPVA/usual	ø	ø	ø	ø	+++	+++	+++	+
14.	36	ECA + CIN2	HPVA/usual	ø	–	–	–	++	+++	++	+
15.	30	ECA	HPVA/usual	–	–	–	–	+++	++	+++	+
16.	31	ECA	HPVA/usual	–	–	–	–	+++	+++	+++	+
17.	90	ECA	HPVA/usual	ø	ø	ø	ø	+++	+++	+++	+
18.	43	ECA	HPVA/usual	ø	ø	–	ø	+++	+++	+++	+
19.	43	ECA	HPVA/usual	ø	ø	ø	ø	+++	+++	+++	+
20.	49	ECA	HPVA/mucinous NOS^7^	ø	ø	–	ø	+++	+++	+++	+
21.	44	ECA	HPVA/usual	ø	ø	ø	ø	+++	+++	+++	+
22.	47	ECA	HPVA/usual	ø	ø	ø	ø	+++	+++	+++	+
23.	32	ECA	HPVA/usual	–	–	–	–	+++	+++	+++	+
24.	42	ECA	HPVA/usual	ø	ø	ø	ø	+++	+++	+++	+
25.	37	ECA	HPVA/usual	–	–	–	–	+++	+++	+++	+
26.	54	ECA	HPVA/usual	–	–	–	–	+++	+++	+++	+
27.	52	ECA	HPVA/usual	ø	ø	–	ø	+++	++	+++	+
28.	50	ECA	HPVA/usual	ø	ø	–	ø	+++	+++	+++	+
29.	36	ECA	HPVA/usual	–	–	–	–	+++	+++	+++	+
30.	86	ECA	HPVA/usual	ø	ø	ø	ø	++	++	++	+
31.	45	ECA	HPVA/usual	ø	ø	–	ø	+++	+++	+++	+
32.	36	ECA + CIN3	HPVA/usual	–	–	–	–	+++	++	+++	+
33.	41	ECA + AIS	HPVA/iSMILE^8^	–	–	–	–	+++	+++	+++	+
34.	48	ECA	NHPVA/serous	–	ø	ø	ø	+++	+++	+++	+
35.	77	ECA	NHPVA/serous	–	–	–	–	+++	+++	+++	+
36.	47	ECA	HPVA/usual	–	–	–	–	+++	+++	+++	+
37.	37	ECA	HPVA/usual	–	–	–	–	+++	+++	+++	+

Endocervical adenocarcinoma

^1^Cervical intraepithelial neoplasia

^2^No normal glandular epithelium present on slide

^3^Immunoreactivity extent: +++ = > 50%, ++ = 10–50%, + = < 10%, − = negative—no obvious immunoreactivity at × 40 magnification

^4^International Endocervical Adenocarcinoma Criteria and Classification [19]

^5^Human papillomavirus–associated adenocarcinoma

^6^Nonhuman papillomavirus–associated adenocarcinoma

^7^Mucinous, not otherwise specified

^8^Invasive stratified mucin–producing carcinoma

^9^Overall score of immunoreactivity evaluation: “−” refers to negative/focally positive (at least two experts rated either “−” or “+”) while “+” refers to robust expression (at least two experts rated either “++” or “+++”)

The detailed results of the EZH2 immunohistochemical analyses for ECA and AIS are summarized in Table 1 (for ECA) and in Table 2 (for AIS).

**Table 2:** Clinical and immunostaining data of AIS cases

Case No.	Age	Diagnosis	EZH2 immunoreactivity^3^							
			Normal glandular epithelium				Neoplastic endocervical lesions			
			Expert			Overall score^4^	Expert			Overall score^4^
			1	2	3		1	2	3	
1.	33	AIS	–	–	–	–	+++	+++	+++	+
2.	53	AIS + CIN1^1^	+	+	–	–	+++	+++	+++	+
3.	42	ECA + AIS	+	–	–	–	+++	+++	+++	+
4.	41	AIS	–	–	–	–	+++	+++	+++	+
5.	38	AIS	–	–	–	–	+++	+++	+++	+
6.	35	AIS	–	–	–	–	+++	+++	+++	+
7.	36	AIS	–	–	–	–	+++	+++	+++	+
8.	31	AIS	–	–	–	–	+++	+++	+++	+
9.	45	AIS + CIN3	ø^2^	ø	–	ø	++	++	++	+
10.	48	AIS + CIN3	–	+	–	–	+++	+++	+++	+
11.	46	AIS + CIN3	–	–	–	–	+++	+++	+++	+
12.	41	AIS + CIN3	–	–	–	–	+++	+++	+++	+
13.	45	AIS + CIN3	–	–	–	–	+++	+++	+++	+
14.	40	AIS + CIN3	–	+	–	–	+++	+++	+++	+
15.	41	ECA + AIS	–	–	–	–	+++	+++	+++	+
16.	38	AIS + CIN3	–	+	–	–	+++	+++	+++	+
17.	65	AIS + CIN3	–	–	–	–	+++	+++	+++	+

Adenocarcinoma in situ

^1^Cervical intraepithelial neoplasia

^2^No normal glandular epithelium present on slide

^3^Immunoreactivity extent: +++ = > 50%, ++ = 10–50%, + = < 10%, − = negative—no obvious immunoreactivity at × 40 magnification

^4^Overall score of immunoreactivity evaluation: “−” refers to negative/focally positive (at least two experts rated either “−” or “+”) while “+” refers to robust expression (at least two experts rated either “++” or “+++”)

All neoplastic endocervical lesions (ECA and AIS) were found to be EZH2 positive by all three experts (for details see Tables 1 and 2). Except for one case out of the 54, all of these lesions (98.14%) received a “robust” overall score.

Adjacent normal glandular epithelium was detected in 34 (63%) out of the 54 malignant cases by at least two experts. On average, immunonegativity was found in 88.3%, while focal positivity in 11.7% of the detected normal glandular epithelium samples by the three experts (for details see Tables 1 and 2). Ratings resulted in an overall score of “negative/focally positive” in all of the 34 cases.

**Fig. 1:**
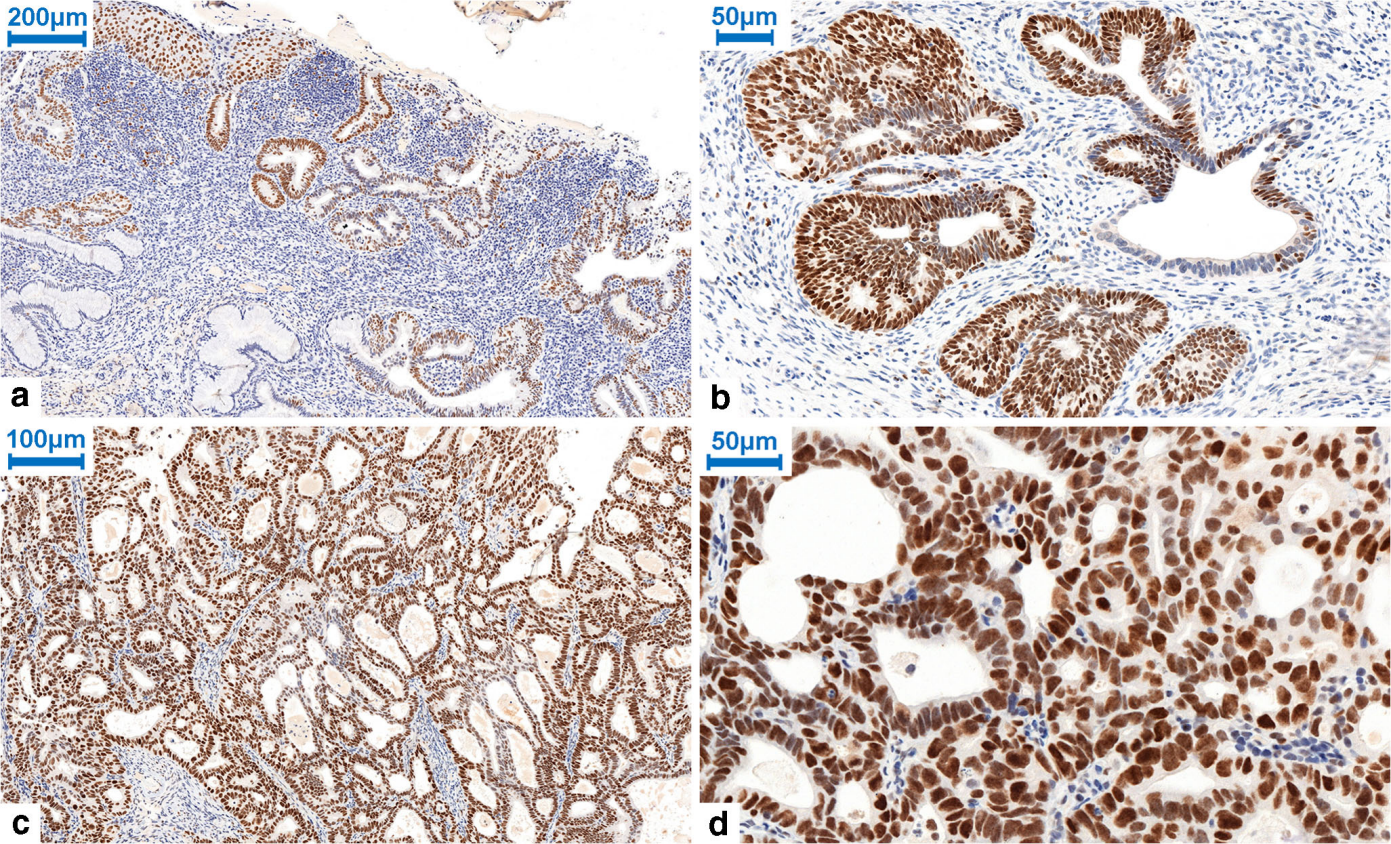
EZH2 nuclear expression in CIN, AIS, and ECA. **a** Diffuse positive (robust) expression of EZH2 in CIN3 and AIS, negative staining of EZH2 in normal endocervical glandules (Table 2; Case No. 11; immunohistochemistry; × 100 magnification). **b** Diffuse positive (robust) expression of EZH2 in AIS (Table 2; Case No. 17; immunohistochemistry; ×300 magnification). **c**–**d** Diffuse positive (robust) nuclear expression of EZH2 in ECA (Table 1; Case No. 22.; immunohistochemistry; × 200 and × 400 magnification)

Figure 1 shows representative cases of diffuse (robust) EZH2 immunoreactivity of the neoplastic endocervical lesions (ECA and AIS) and negative immunoreactivity of the adjacent normal glandular epithelium.

A total of 32 non-neoplastic endocervical lesions (15 reactive atypia, 9 microglandular hyperplasia, 3 tuboendometrioid metaplasia, 3 tunnel cluster, 2 endometriosis) were analyzed.

The detailed results of the EZH2 immunohistochemical analyses for non-neoplastic endocervical lesions were summarized in Table 3.

**Table 3:** Clinical and immunostaining data of non-neoplastic cases

Case No.	Age	Diagnosis	EZH2 immunoreactivity^2^							
			Normal glandular epithelium				Non-neoplastic endocervical lesions			
			Expert			Overall score^3^	Expert			Overall score^3^
			1	2	3		1	2	3	
1.	33	Reactive atypia	–	–	–	–	–	–	–	–
2.	50		–	–	–	–	+	+	–	–
3.	39		–	–	–	–	–	–	–	–
4.	54		–	–	–	–	+	+	+	–
5.	40		–	–	–	–	+	+	–	–
6.	40		–	–	–	–	–	–	–	–
7.	42		–	–	–	–	–	–	–	–
8.	39		++	–	–	–	++	++	+	+
9.	49		++	–	–	–	++	++	+	+
10.	43		–	–	+	–	+	+	+	–
11.	32		–	–	–	–	–	–	–	–
12.	46		–	–	–	–	+	–	+	–
13.	61		–	–	–	–	–	–	–	–
14.	48		–	–	–	–	–	–	–	–
15.	48		–	–	–	–	–	–	–	–
16.	51	Microglandular hyperplasia	–	–	+	–	–	–	–	–
17.	50		–	–	–	–	–	–	–	–
18.	37		–	–	–	–	–	–	–	–
19.	69		–	–	–	–	+	+	+	–
20.	37		–	–	–	–	–	–	–	–
21.	54		–	–	–	–	+	+	+	–
22.	37		–	–	–	–	–	–	–	–
23.	48		–	–	–	–	–	–	+	–
24.	51		–	–	–	–	–	–	–	–
25.	35	Endometriosis	–	–	–	–	–	–	–	–
26.	33		–	–	–	–	–	–	–	–
27.	36	TEM^1^	–	–	–	–	–	–	–	–
28.	41		–	–	–	–	++	++	+	+
29.	46		–	–	–	–	–	–	–	–
30.	61	Tunnel cluster	–	–	–	–	–	–	–	–
31.	53		–	–	–	–	++	++	+	+
32.	67		–	–	–	–	–	–	–	–

^1^Tuboendometrioid metaplasia

^2^Immunoreactivity extent: ++ = 10–50%, + = < 10%, − = negative—no obvious immunoreactivity at × 40 magnification

^3^Overall score of immunoreactivity evaluation: “−” refers to negative/focally positive (at least two experts rated either “−” or “+”) while “+” refers to robust expression (at least two experts rated either “++” or “+++”)

On average, 67.7% of the ratings were negative, 24% of the ratings were focally positive, and 8.3% of the ratings were partly positive. The ratings resulted in an overall score of “negative/focally positive” in 28 out of the 32 cases (87.5%) and “robust” in the rest of the cases (4 cases, 12.5%).

Adjacent normal glandular epithelium was detected in all non-neoplastic endocervical lesion cases by all experts.

**Fig. 2:**
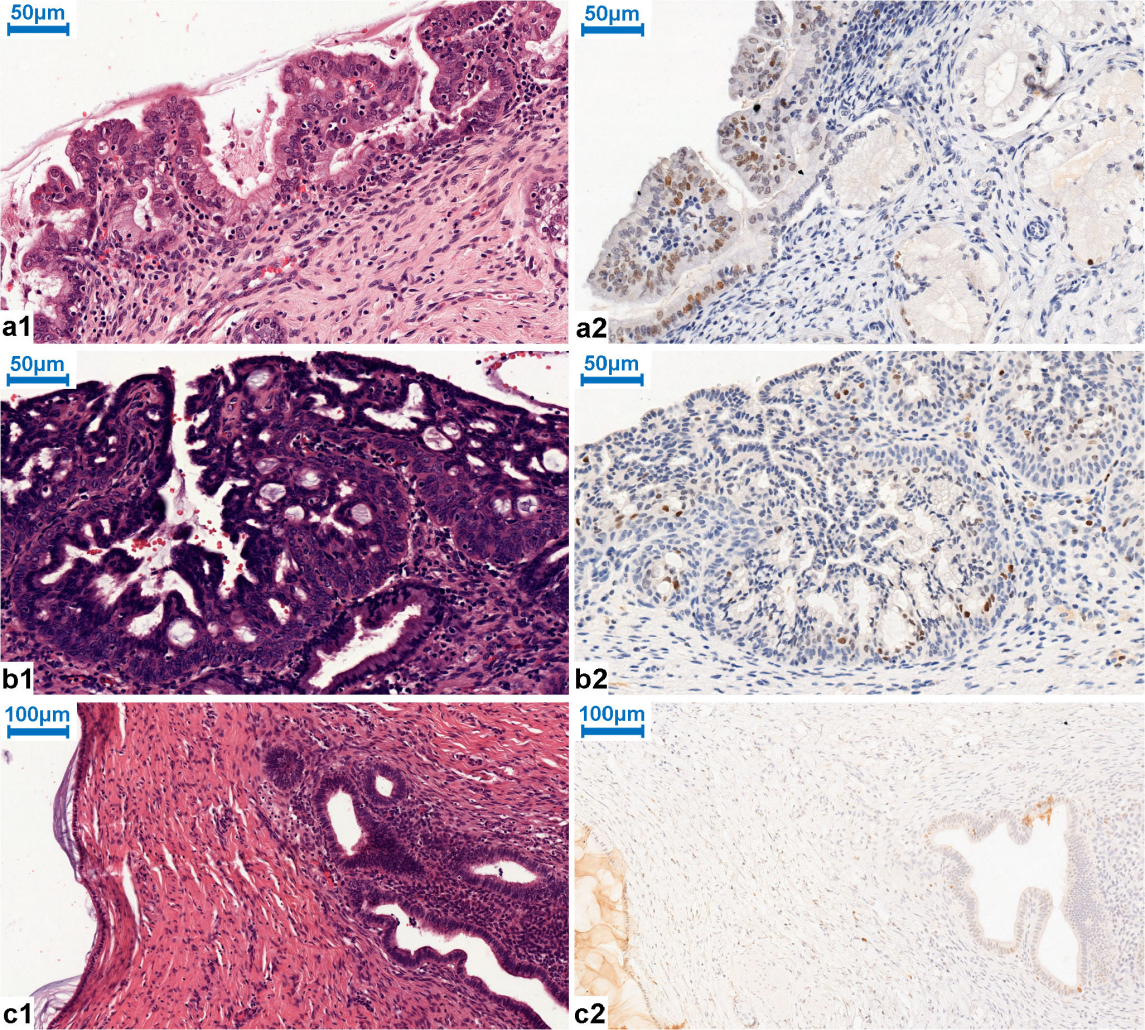
Reactive atypia, microglandular hyperplasia, and endometriosis with HE. a1, b1 (× 400 magnification), c1 (× 200 magnification). a2 EZH2 focal positivity in reactive atypia (Table 3; Case No. 10; immunohistochemistry; × 400 magnification). b2 No expression of EZH2 in microglandular hyperplasia, EZH2 focal positivity in squamous metaplasia (Table 3; Case No. 24; immunohistochemistry; × 400 magnification). c2 Negative staining of EZH2 in endometriosis (Table 3; Case No. 25; immunohistochemistry; × 200 magnification)

**Fig. 3:**
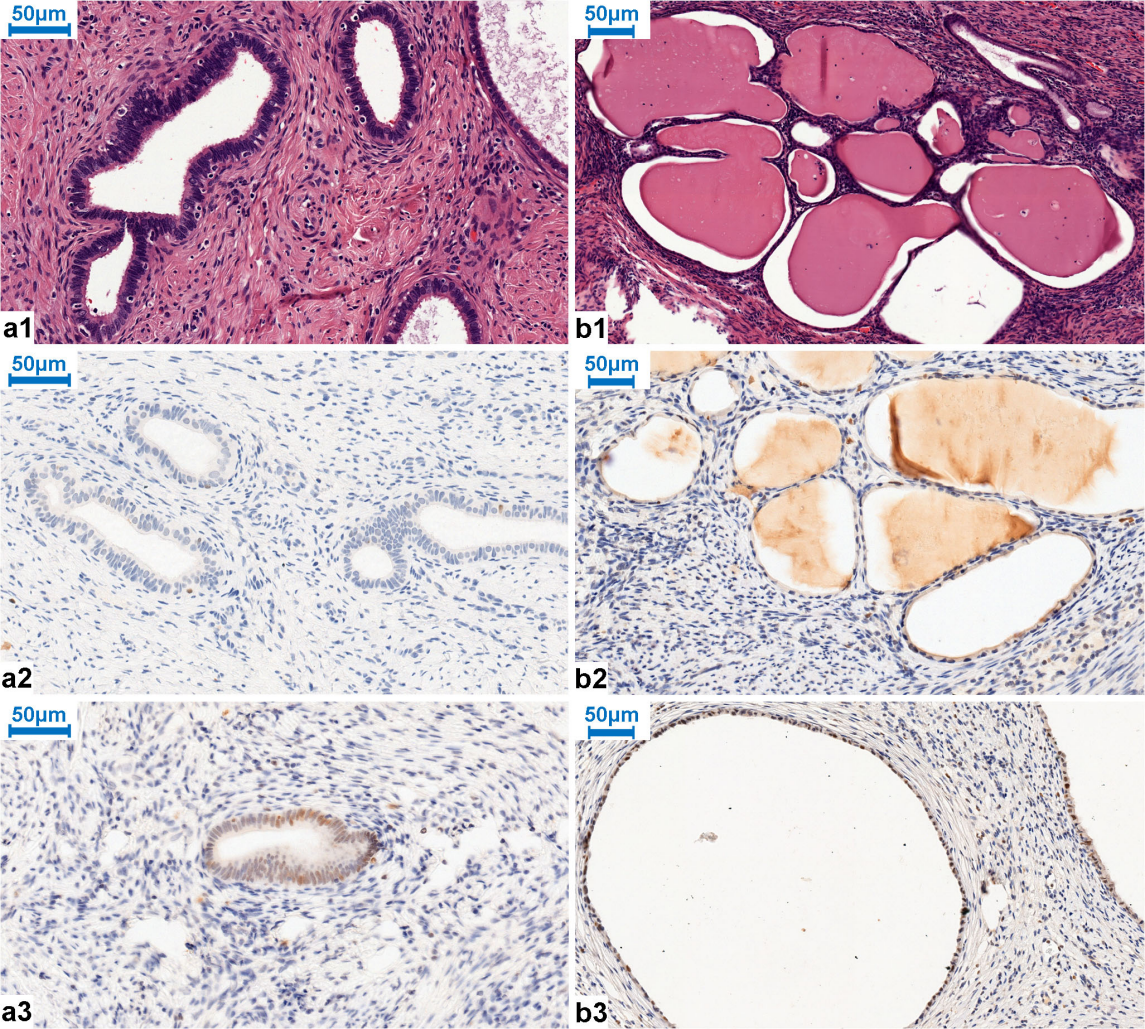
Tuboendometrioid metaplasia (TEM) and tunnel cluster with HE. a1, b1 (a1, × 400 magnification; b1 × 300 magnification). a2 EZH2 negative staining in TEM (Table 3; Case No. 27; × 400 magnification). a3 Partly positive (robust) expression (++) of EZH2 in another sample with TEM (Table 3; Case No. 28; × 400 magnification). b2 EZH2 negativity in tunnel cluster (Table 3; Case No. 30; × 300 magnification). b3 Partly positive (robust) expression (++) of EZH2 in another case of tunnel cluster (Table 3; Case No. 31; × 300 magnification)

These adjacent normal glandular epithelium samples were on average rated immunonegative in 95.84%, focally positive in 2.08%, and partially positive in other 2.08% (for details see Table 3). These ratings resulted in an overall score of “negative/focally positive” for each sample. Figures 2 and 3 show representative cases of negative or focally positive (+) EZH2 immunoreactivity of the non-neoplastic lesions.

Fisher’s exact test yielded a statistically significant (two-tailed *p* < 0.0001) difference in the overall immunoreactivity scores between the neoplastic and non-neoplastic lesions (“robust” score was found in 53 out of the 54 neoplastic lesions vs. in 4 out of the 32 non-neoplastic lesions).

Robust EZH2 expression was found to have a sensitivity of 98.15% (95% CI = 90.11 to 99.95%) and a specificity of 87.5% (95% CI = 71.01 to 96.49%) in distinguishing neoplastic lesions from non-neoplastic lesions, with a positive predictive value of 92.98% (95% CI = 83 to 98.05%) and a negative predictive value of 96.55% (95% CI = 82.24 to 99.91%). A sensitivity of 98.15% (95% CI = 90.11 to 99.95%) and a specificity of 100% (95% CI = 94.4 to 100%) were found in distinguishing neoplastic lesions from all normal glandular epithelium samples (*n* = 66), with a positive predictive value of 100% (95% CI = 93.28 to 100%) and a negative predictive value of 98.46% (95% CI = 91.72 to 99.96%). A sensitivity of 98.15% (95% CI = 90.11 to 99.95%) and a specificity of 95.88% (95% CI = 89.78% to 98.87%) were found in distinguishing neoplastic from non-neoplastic lesions and normal endocervical epithelium samples combined (*n* = 98), with a positive predictive value of 92.98% (95% CI = 83 to 98.05%) and a negative predictive value of 98.46% (95% CI = 94.17 to 99.97%).

For the neoplastic endocervical lesion (ECA and AIS) immunoreactivity ratings, inter-expert ICCs were 0.53 for single measures (95% confidence interval = 0.37–0.67) and 0.77 for average measures (95% confidence interval = 0.64–0.86).

For the non-neoplastic endocervical lesion immunoreactivity ratings, inter-expert ICCs were 0.8 for single measures (95% confidence interval = 0.68–0.89) and 0.92 for average measures (95% confidence interval = 0.86–0.96).

## Discussion

The aim of this study was to investigate the EZH2 expression status of neoplastic endocervical lesions such as ECA and AIS compared with normal glandular epithelium and non-neoplastic endocervical lesions.

All endocervical neoplastic lesions in this study were found to be EZH2 positive by all experts. Moreover, immunoreactivity was found to be very extensive. Except for one case, all (98.14%) neoplastic lesions showed a robust EZH2 expression.

In contrast, robust EZH2 expression was significantly less often (4 out of 32 cases, 12.5%) found in the non-neoplastic glandular lesions (two-tailed *p* < 0.0001) and never (0 out of 66 samples) in the adjacent normal glandular epithelium. Occasionally, false positivity was caused by squamous metaplasia or reserve cell hyperplasia (e.g., in Fig. 2 case b).

Robust EZH2 expression appeared to have an excellent diagnostic test capability in differentiating neoplastic lesions from non-neoplastic lesions and normal endocervix. A sensitivity of 98.15% and a specificity of 95.88% were found in distinguishing neoplastic from non-neoplastic lesions and normal endocervical epithelium samples combined (*n* = 98), with a positive predictive value of 92.98% and a negative predictive value of 98.46%.

Inter-observer agreement for average measurements could be interpreted as excellent [23].

Our presented data suggest that EZH2 plays a role in the pathogenesis of not only malignancies of the breast [7], lung [8], stomach [9], colon [10], pancreatobiliary tract [11], liver [12], thyroid gland [13], prostate [14], bladder [15], endometrium [16], and ovary [17] but also in endocervical neoplasia as well. Since EZH2 expression was found in all investigated cases including the non-human papillomavirus–related ones, authors speculate that EZH2 is a substantial and independent factor in endocervical carcinogenesis.

Yuting Gu et al. [24] studied the expression of EZH2 in endometrial carcinomas. The expression rate of EZH2 in endometrial carcinoma tissue (68.27%) was significantly higher than that in adjacent tissue (24.03%). Nan Jia et al. [25] demonstrated that EZH2 was overexpressed (medium to strong reactivity) in complex hyperplasia, atypical hyperplasia, and endometrial cancer, but not in simple hyperplasia and normal endometrium (with negative to weak expression). In the study by Jin et al., aberrant overexpression of EZH2 was frequently observed in cervical squamous cell carcinoma as compared with adjacent normal tissues (*P* = 0.0005). Although these studies investigated immunoreactivity intensity, unlike immunoreactivity extent as in the present study, the results appear to be still comparable. EZH2 immunoreactivity differences between neoplastic and non-neoplastic and/or normal tissues appear to be at least as appreciable in the endocervix as in the endometrium or cervical squamous epithelium. This raises that EZH2 staining might be applied as a differential diagnostic tool in endocervical lesions. At present, panels including combinations of various markers are suggested for endocervical differential diagnosis. Sandra Lee et al. [26] showed that p16, p16/Ki67 dual stain, ProExC, CEA, ESA, HIK1083, Claudin 18, and ER losses in perilesional stromal cells were useful with high (≥ 0.75) sensitivity and specificity estimates in ≥ 1 malignant versus benign comparisons. Our data indicate that robust expression of EZH2 alone has an even higher diagnostic reliability, with a sensitivity and specificity of over 95%.

As a conclusion, EZH2 may play a role in the pathogenesis of endocervical neoplasia, and the detection of robust expression of EZH2 might be a useful differential diagnostic tool in problematic endocervical lesions in histology and probably in cytology as well.
